# *Helicobacter pylori* Antibiotic Resistance: Molecular Basis and Diagnostic Methods

**DOI:** 10.3390/ijms24119433

**Published:** 2023-05-29

**Authors:** Irina Medakina, Larisa Tsapkova, Vera Polyakova, Sergey Nikolaev, Tatyana Yanova, Natalia Dekhnich, Igor Khatkov, Dmitry Bordin, Natalia Bodunova

**Affiliations:** 1 SBHI Moscow Clinical Scientific Center, 111123 Moscow, Russia; 2 FSBEI HE Smolensk State Medical University of the Ministry of Health of Russia, 214019 Smolensk, Russia; 3 Department of Propaedeutic of Internal Diseases and Gastroenterology, FSBEI HE Moscow State University of Medicine and Dentistry, 127473 Moscow, Russia; 4 Department of General Medical Practice and Family Medicine, FSBEI HE Tver State Medical University of the Ministry of Health of Russia, 170100 Tver, Russia

**Keywords:** *Helicobacter pylori*, antibiotic resistance, methods for determining *H. pylori* resistance, molecular genetic diagnostics, phenotypic methods for determining antibiotic resistance, geographical distribution of *H. pylori* antibiotic resistance

## Abstract

*Helicobacter pylori* is one of the most common cause of human infections. Infected patients develop chronic active gastritis in all cases, which can lead to peptic ulcer, atrophic gastritis, gastric cancer and gastric MALT-lymphoma. The prevalence of *H. pylori* infection in the population has regional characteristics and can reach 80%. Constantly increasing antibiotic resistance of *H. pylori* is a major cause of treatment failure and a major problem. According to the VI Maastricht Consensus, two main strategies for choosing eradication therapy are recommended: individualized based on evaluating sensitivity to antibacterial drugs (phenotypic or molecular genetic method) prior to their appointment, and empirical, which takes into account data on local *H. pylori* resistance to clarithromycin and monitoring effectiveness schemes in the region. Therefore, the determination of *H. pylori* resistance to antibiotics, especially clarithromycin, prior to choosing therapeutic strategy is extremely important for the implementation of these treatment regimens.

## 1. Introduction

*Helicobacter pylori* is one of the most common human infections [1,2,3]. Infected patients develop chronic active gastritis in all cases, which can lead to peptic ulcer, atrophic gastritis, gastric cancer and gastric MALT-lymphoma [4,5,6,7]. Eradication of *H. pylori* can prevent long-term complications, or relapses of the disease. The treatment the infection is recognized as the primary prevention of gastric cancer.

The prevalence of *H. pylori* infection in the population has geographical distribution characteristics, determined by the level of hygiene, and can reach 80% [8]. According to a meta-analysis published in 2017, the number of people infected with *H. pylori* in the Russian Federation was estimated at 78.5% [9]. At the same time, the prevalence of infection in developed countries is decreasing. Thus, a recently published study showed, that about 40% of the population in Russia is infected, while the prevalence of *H. pylori* increases with age [10].

The recently published Maastricht VI Consensus proposed two strategies for selecting eradication therapy: individualized, based on antibacterial susceptibility, and empirical, in which data regarding local resistance of *H. pylori* to clarithromycin (<15% or >15%) are taken into account when choosing a treatment regimen and monitoring the effectiveness of schemes used in the particular region [4]. Therefore, the determination of *H. pylori* resistance to antibiotics, primarily to clarithromycin, is extremely important for the implementation of these treatment strategies.

The efficacy of antibacterial drugs depends on the sensitivity of the bacteria to them. Currently, a major problem is the decreasing efficacy of therapy regimens, which is associated with the rise of antibiotic resistance in *H. pylori* strains. The prevalence of *H. pylori* resistance varies in different countries and depends on the overall frequency of antibiotic use.

The main objective of this study was to provide a comprehensive analysis of current state of research on the study of antibiotic resistance in *H. pylori*, the molecular mechanisms underlying its emergence, as well as an overview and comparison of methods used for resistance evaluation.

**Figure 1:**
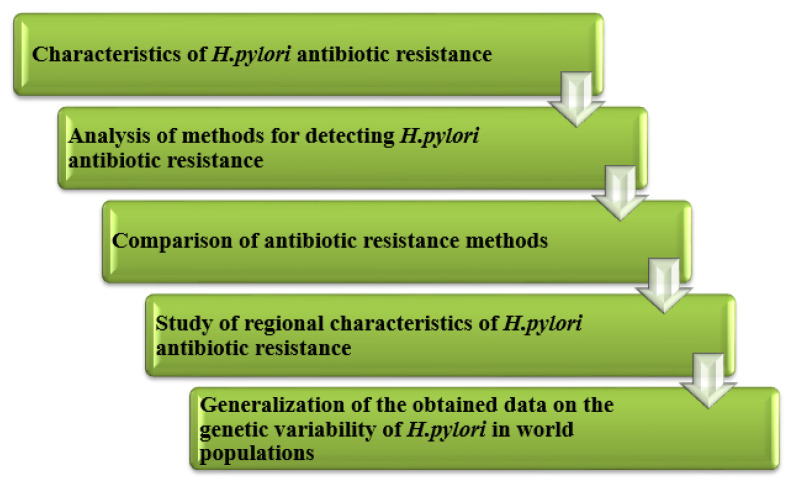
The structure of this article.

The structure of this article is presented in a diagram (Figure 1).

## 2. Characteristics of *H. pylori* Antibiotic Resistance

It has been previously demonstrated, that antibiotic resistance in *H. pylori* a result of mutations in its genome. Point mutations in the V domain of *23S* ribosomal RNA can change the affinity of clarithromycin for the peptidyltransferase loop and lead to resistance to clarithromycin [11,12,13]. The most common *23S* rRNA mutations associated with clarithromycin resistance are A2143G, A2142G, A2142C [11,12,13]. The sensitivity of *H. pylori* to clarithromycin is also affected by outer membrane proteins. Proteins such as HopT (BabB), HofC, and OMP31 were absent in clarithromycin-sensitive strains, and were only identified in clarithromycin-resistant strains of *H. pylori*. The mechanism of their association with antibiotic resistance is not yet to metronidazole resistance clear [11].

Mutations in the *rdxA* gene encoding oxygen-insensitive nitroreductase and in the *frxA* gene encoding flavin oxidase reductase are the main cause of resistance to metronidazole. Mutations in these genes reduce the ability of metronidazole to recover to active forms (NO^2−^, NO_2_^2−^), which have a damaging effect on the bacterial DNA structure [14,15].

Levofloxacin is a fluoroquinolone, which acts via its interaction with DNA gyrase encoded by the *gyrA* and *gyrB* genes. DNA gyrase performs an important function: it promotes the separation of DNA strands during replication. Under the influence of levofloxacin, the process of DNA synthesis and the process of replication of a bacterial cell are disrupted. In levofloxacin-resistant strains of *H. pylori*, mutations were found in codons 87, 88, 91, 97 of the *gyrA* gene and in position 463 of the *gyrB* gene [11,12,13].

Amoxicillin belongs to the group of beta-lactams, interacts with penicillin-binding proteins and leads to the disruption of cell wall synthesis and destruction of *H. pylori*. Mutations that can interfere with cell wall synthesis disrupt the mechanism of action of amoxicillin: *pbp1A*, *pbp2*, *pbp3*, *hefC*, *hopC* and *hofH*. In addition, the effect of amoxicillin on *H. pylori* is further complicated by the fact that the bacterium itself produces beta-lactamases and there is a reduced membrane permeability for amoxicillin due to efflux pumps [16,17,18].

In a cohort of Taiwanese patients (*n* = 70) diagnosed with refractory *H. pylori* infection, 39 isolates were successfully cultured. *H. pylori* isolates were obtained from the gastric mucosa, subjected to phenotypic testing for sensitivity to amoxicillin and molecular analysis of genetic variants of the *pbp1A* amoxicillin resistance gene. 30 substitutions were identified (K352, K363, F366, G367, A369, V374, Q376, T386, F396, H409, S414, R418, F448, F473, D508, V509, T513, L530, T541, S543, T550, N561, G59 1, Y604, S615, K617, R618, F620, V622 and P623) in amoxicillin-resistant isolates. It is noted that the majority of resistant isolates carry the P623L substitution, which is potentially responsible for the development of resistance to amoxicillin. Three amino acid substitutions (D479E, D535N and S589G) were identified in all amoxicillin-resistant *H. pylori* isolates obtained from Taiwanese patients [19].

Tetracycline, an antibiotic from the tetracycline group, destroys the codon-anticodon bond at the level of the 30S ribosome subunit, which halts bacterial protein synthesis. Mutations in the *16S rRNA* of the *TET-1* gene lead to the development of resistance to tetracycline. The most common genetic alteration is the substitution of the AGA-GGA triplet (926–928) [20].

The bactericidal action of rifabutin is realized due to its interaction with DNA-dependent RNA polymerase and leads to inhibition of the process of transcription of bacterial DNA. Resistance to rifabutin arises due to mutation of the *rpoB* gene, which encodes the beta subunits of RNA polymerase [1].

Furazolidone (nitrofuran), affects the activity of bacterial oxidoreductase, thus disrupting bacterial metabolism. Mutations associated with resistance to this antibiotic have been identified in the *porD* and *oorD* genes encoding integral ferredoxin-like subunits [21].

Despite the fact that the factors ensuring the adaptation of *H. pylori* to antibiotics are already known, in particular: the effect of the efflux pump, membrane permeability, changes in outer membrane proteins, the ability to form a biofilm and mutations in number of genes, the molecular mechanisms of some remain unclear. Thus, the matter of prescribing effective therapy against *H. pylori* for patients with antibiotic resistance to several drugs remains open [1,22].

## 3. Methods for Detection of *H. pylori* Antibiotic Resistance

The methods used to detect antibiotic resistance are divided into phenotypic and molecular genetic. Phenotypic methods include: diffusion method (disk diffusion), serial dilution method (in agar, in broth) and combined E-test [23,24,25,26,27,28].

The method of serial dilutions (limiting dilutions) is based on the determination of a quantitative indicator characterizing the microbiological activity of an antibiotic–the minimum inhibitory concentration (MIC). To determine the MIC, concentrations of the drug prepared in advance are added to the nutrient medium, followed by inoculation and incubation of nutrient medium with the antibiotic. After incubation, bacterial growth is assessed and the active dose of the antibiotic is determined [26,27]. Depending on the concentrations to which *H. pylori* strains are sensitive, they can be divided into sensitive, moderately resistant and resistant. The dilution method using liquid nutrient media is quite convenient and it can be automated, however, due to the difficulties of cultivating *H. pylori*, this method is not convenient for large-scale application.

The disk-diffusion method is based on the diffusion of the drug from the carrier into a dense nutrient medium and suppression of the growth of the studied culture in the zone where the antibiotic concentration exceeds the minimum inhibitory concentration [28]. A paper disk is used as an antibiotic carrier in the disk diffusion method. The effect of an antibiotic is estimated by the diameter size of the growth suppression zone. The growth inhibition diameter, the lower the MIC of the antibiotic and the more active it is against the microorganism under study. Due to the presence of a sufficiently long period of time between the preparation of the medium and the start of this test, the result may be distorted due to a change in the redox potential of the medium, which makes it impossible to use this method for the study of metronidazole. It should be noted that there are no clear criteria for interpreting the results obtained from this method; therefore, at present, the disk diffusion method is not practically used.

The E-test is a type of the disk-diffusion method, where a polymer strip is used as an antibiotic carrier, on which a drug concentration gradient is applied. The activity of the antibiotic is also assessed by the inhibition zone of the microorganisms’ growth–the drop-shaped zone of growth inhibition. The MIC value is assessed in the place where the zone of growth suppression is closely adjacent to the drug carrier [23,24,25].

Criteria for assessing the sensitivity of *H. pylori* to antimicrobial agents are shown in Table 1.

Molecular genetic methods for detecting antibiotic resistance include real-time polymerase chain reaction (RT-PCR), hybridization with oligonucleotide probes, analysis of restriction fragment length polymorphism (RFLP), Sanger sequencing and NGS sequencing. These methods facilitate the identification of specific mutations that lead to antibiotic resistance.

The PCR-RFLP method is based on amplification of DNA gene regions and selective cleavage of PCR products using restriction endonucleases that recognize mutation sites.

Real-time PCR is the most sensitive and specific method for the detection of infectious agents in comparison with traditional methods (phenotypic, immunological). Real-time polymerase chain reaction is a repetitive cycle of target DNA synthesis. In each cycle, the number of copies of the amplified region is doubled, which makes it possible to generate a DNA fragment bounded by a pair of selected primers in an amount sufficient for its detection using fluorescent probes in 35 cycles. By the end of the procedure, at least 1012 fragments should accumulate. The real-time PCR method allows both to determine the presence of a pathogen and its quantification it. Since the kinetics of amplicon accumulation directly depends on the number of the evaluated matrix copies, which allows quantitative measurements of DNA and RNA of infectious agents. To date, real-time polymerase chain reaction method is characterized by one of the lowest error rates [29]. A group of scientists led by M. Pichon conducted a study of *H. pylori* resistance to clarithromycin in stool samples using real-time PCR. Sensitivity and specificity for detecting *H. pylori* were 96.3% (95% CI, 92–98%) and 98.7% (95% CI, 97–99%), respectively [30].

Fluorescence in situ hybridization (FISH) is used to detect *H. pylori* antibiotic resistance in biopsy specimens. The method is applicable to histological preparations. The interpretation of the results is carried out using a fluorescent microscope.

The Sanger sequencing method is the analysis of the studied DNA section’s nucleotide sequence of the, namely, the determination of the exact order of the nucleotides in the DNA molecule. With its help, in one working cycle, it is possible to “read” sequences up to 1000 base pairs long with a high accuracy of 98% [31].

NGS–next generation sequencing. The main advantage of the method is high performance and accuracy of the method. Modern sequencers have a capacity of more than 15 billion base pairs per run, maximum read length of more than 600 base pairs and ability to analyze up to 96 samples per run. However, this method is very expensive and requires highly qualified personnel. Therefore, its implementation into clinical practice remains problematic. It is possible that in the future, with the reduction in the cost of analysis, NGS would be implemented into routine clinical practice [32].

A significant advantage of molecular genetic methods in comparison to phenotypic methods is their automation, lower labor input, and high accuracy of results.

Hulten K.G. with a group of scientists compared molecular genetic (NGS) and phenotypic methods for determining the sensitivity of *H. pylori* to amoxicillin, clarithromycin, metronidazole, levofloxacin, tetracycline and rifabutin. The NGS analysis was aimed at studying the already known antimicrobial resistance genes *23SrRNA*, *gyrA, 16SrRNA*, *pbp1*, *rpoB*, and *rdxA*. The study was carried out on tissue samples of the gastric mucosa fixed in formalin. The results showed that compared to the phenotypic method, the NGS method determined resistance to clarithromycin, levofloxacin, rifabutin and tetracycline with higher accuracy. However, the results for amoxicillin and metronidazole were noted to be less accurate. This fact might be attributed to the poorly understood molecular characteristics of this antibiotic resistance type. There is a possibility that genetic changes in other genes may also be associated with resistance to metronidazole and amoxicillin [33].

The results of recent study regarding the evaluation of *H. pylori* resistance to antibiotics demonstrate a close correlation between the results of NGS analysis of stool samples and gastric biopsy samples from the same patients, which indicates the suitability of feces as a biological material for molecular genetic identification of *H. pylori* antibiotic resistance [34]. Comparative characteristics of *H. pylori* antibiotic resistance research methods are presented in Table 2.

Interesting findings have been reported in a review paper by Francesca Celiberto et al. on a molecular genetic study of *H. pylori* antibiotic resistance in stool samples. The authors analyzed scientific publications for the period from 1996. The results of these studies showed high sensitivity, and majority of them–high specificity, of the molecular method for determining *H. pylori* antibiotic resistance in stool samples compared to phenotypic methods and RT-PCR of gastric biopsies. Interestingly, the method proved to be more reliable in diagnosing the infection in comparison with commonly used non-invasive diagnostic methods: 13C-urease breath test and fecal antigen determination [35].

## 4. Regional Characteristics of *H. pylori* Resistance

**Figure 2:**
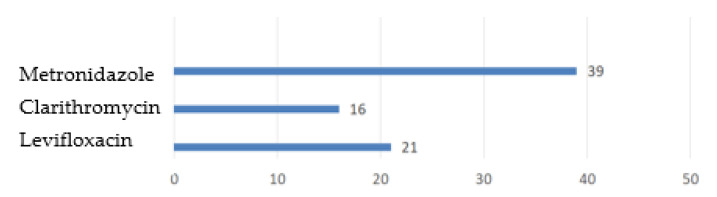
Antibiotic resistance rates (in %) in Europe.

*H. pylori* has regional resistance patterns. In Europe, resistance rates of *H. pylori* to clarithromycin are 18–21.4%, levofloxacin −11.0–16.3%, to metronidazole −39.1–56%. At the same time, in the countries of Central and Southern Europe these rates are significantly higher in comparison to the countries of Northern Europe (Figure 2) [36].

**Figure 3:**
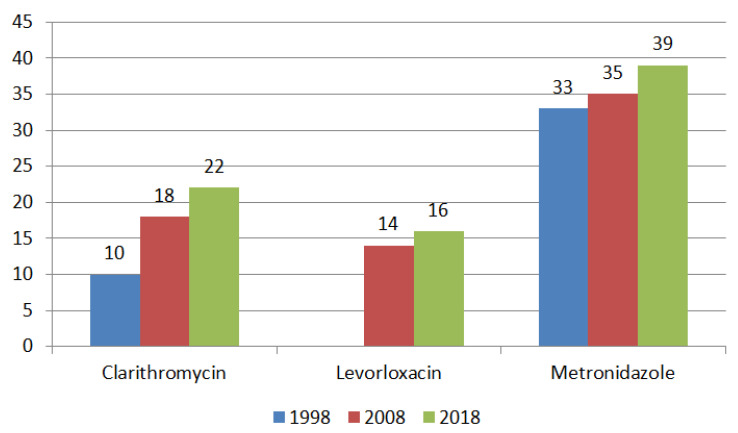
Dynamics of H. pylori antibiotic resistance (%) in Europe. 1998: *n* = 1227 (22 centers, 17 countries); 2008: *n* = 1893 (32 centers, 18 countries); 2018: *n* = 1332 (24 centers, 18 countries).

There has been an increase in *H. pylori* resistance to clarithromycin, levofloxacin, and metronidazole in Europe (Figure 3).

In the USA, resistance to clarithromycin has been reported at the level of 10% [37]. In China, primary *H. pylori* resistance to clarithromycin, metronidazole, and levofloxacin is estimated at 28.9%, 63.8%, and 28%, respectively. Similar rates of *H. pylori* resistance are observed in South Korea [38,39].

**Figure 4:**
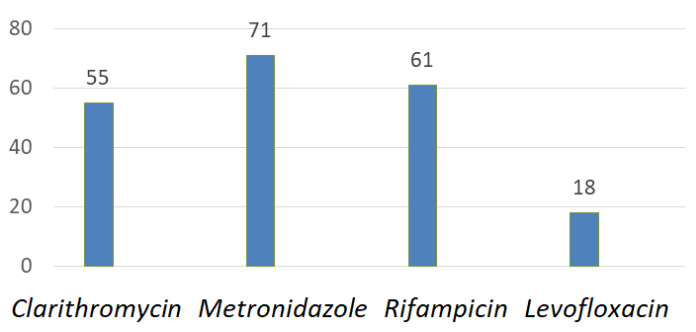
Antibiotic resistance rates (%) of *H. pylori* in Southwest China.

General rates of antibiotic resistance in Southwest China are shown in Figure 4 [40].

**Figure 5:**
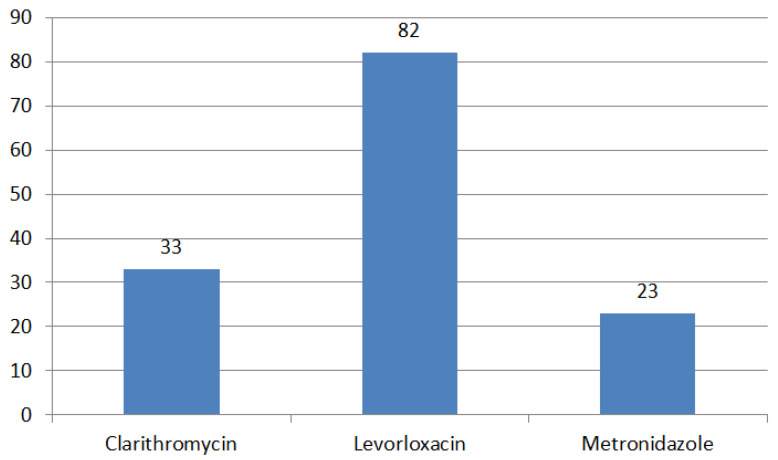
*H. pylori* antibiotic resistance rates (%) in children in Southeast China.

A study was conducted to investigate the changes in antibiotic resistance over time among children in southeastern China. *H. pylori* was cultured from gastric biopsies obtained from children in the time period from 2015 to 2020. Sensitivity to clarithromycin (CLA), amoxicillin (AML), metronidazole (MTZ), furazolidone (FZD), tetracycline (TET), and levofloxacin (LEV) was evaluated. Previously reported data from 2012 to 2014 was used to compare temporal trends in antibiotic resistance. A total of 1638 *H. pylori* strains (52.7%) were isolated from biopsies of 3111 children. The resistance rates to CLA, MTZ, and LEV were 32.8%, 81.7%, and 22.8%, respectively (Figure 5). Single resistance was found in 52.9% of strains, double resistance in 28.7%, and triple resistance in 9.0%. The overall resistance rate and resistance rates to CLA, MTZ, LEV, CLA + LEV, and CLA + MTZ + LEV increased linearly every year. All types of resistance, except single resistance, clearly increased from 2015 to 2017 and from 2018 to 2020 compared with 2012–2014. Double resistance to CLA + MTZ increased significantly with age. The resistance rate to CLA and triple resistance to CLA, MTZ, and LEV were higher in children with previously treated for *H. pylori* infection in comparison to the ones who did not receive any treatment. The rates of antibiotic resistance of *H. pylori* were found to be at high levels in a large cohort of children in southeastern China from 2015 to 2020 [41].

**Figure 6:**
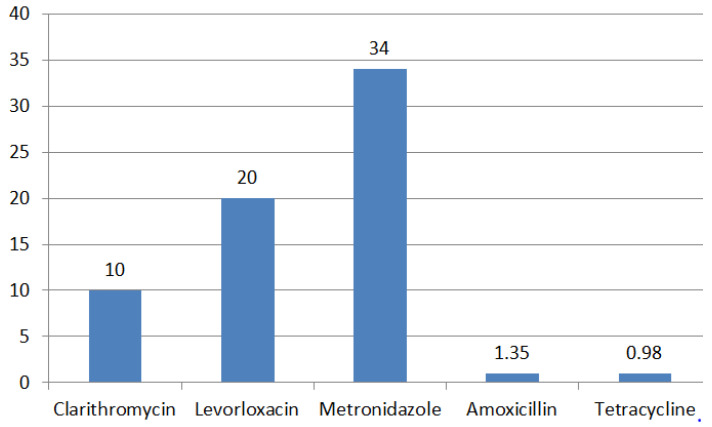
Antibiotic resistance rates (%) in Russia.

The number of *H. pylori* strains studied in Russia is limited and most studies were carried out about 10 years ago. According to a recently published meta-analysis, in Russia, the level of *H. pylori* resistance to clarithromycin is 10%, to levofloxacin—20%, metronidazole—34%, amoxicillin—1.35% and tetracycline—0.98% (95% CI) (Figure 6) [42].

Data on the low resistance of *H. pylori* to clarithromycin contradicts the data obtained from clinical practice, reflected in the European register of *H. pylori* infection management (Hp-EuReg). According to Hp-EuReg the effectiveness of classical triple therapy in Russia is only 80% with a prescription frequency of 56% [43]. Based on the data from the European registry Hp-EuReg, resistance of *H. pylori* to clarithromycin in Russia is 24%, to levofloxacin—27%, to metronidazole—29% [44].

During the revision of our article, a review paper on the evolution of *H. pylori* resistance to antibiotics by Lyudmila Boyanova was published [45]. The study included review articles on antibiotic resistance data from 14 countries, including Australia, Belgium, Bulgaria, Chile, China, Colombia, France, Italy, Iran, Russia, Spain, Taiwan, Vietnam, and the United States. The most commonly used susceptibility test in these studies was the E-test, the agar dilution method, disk diffusion whereas molecular methods were less common. Sensitivity criteria according to EUCAST and CLSI were used. The dynamics of resistance to amoxicillin, metronidazole, tetracycline, levofloxacin were evaluated. It has been shown that in some countries, such as Bulgaria, Belgium, Iran and Taiwan, there has been an increase in *H. pylori* resistance to three or more antibacterial drugs over time, in France and Spain, on the contrary, resistance levels to most antibiotics have stabilized. The absence of antibiotic resistance growth and even a decrease in resistance levels were usually associated with a decrease in the consumption of that antibiotic in the country, adherence to the latest recommendations for the treatment of *H. pylori* infections, and strict antibiotic policies in countries such as France and the United States [46,47].

Table 3 presents a summary of studies regarding *H. pylori* genetic resistance in world populations. Colombia and South America are characterized by relatively high rates of *H. pylori* infection and stomach cancer. Antibiotic resistance was evaluated for 28 strains of *H. pylori* isolated from gastric biopsy samples from residents of two Colombia regions: with a high risk of gastric cancer (HGCR), and 31 strains from a region with a low risk of gastric cancer (LGCR). Mutations leading to antibiotic resistance were investigated by PCR for all isolates, and for 29 isolates whole genome sequencing was performed. None of the strains were resistant to amoxicillin, clarithromycin, or rifampin. One strain was resistant to tetracycline and had the A926G mutation in the *16S rRNA* gene. Levofloxacin resistance was observed in 12 of 59 isolates and was mainly associated with N87I/K and/or D91G/Y mutations in *gyrA*. Most of the isolates were resistant to metronidazole, and this resistance was significantly higher in the low-risk gastric cancer group (31/31) compared to the high-risk gastric cancer group (24/28). Mutations in the *rdxA* and *frxA* genes were present in almost all metronidazole-resistant strains [48].

Sequencing of the *H. pylori 23S rRNA* gene in the Iranian population showed that the most common mutations leading to antibiotic resistance to clarithromycin are the A2143G and A2142 mutations [49].

During the analysis of the *rdxA* and *frxA* gene in 12 metronidazole-resistant and 10 metronidazole-sensitive *H. pylori* strains in the Myanmar population, it was found that all twelve resistant strains had mutations in the *rdxA* gene, three of them contained mutations with a preliminary stop codon. The most common was the point substitution V175I (8/12, 66.7%), followed by S91P (5/12, 41.7%) and R16H/C (4/12, 33.3%). Mutations in the *frxA* gene were observed in 76.9% (10/13) of resistant strains, preliminary early stop codon was observed in only one strain. In the *frxA* gene, the most frequent point mutation was the L33M substitution (3/13, 23.1%) [50].

Several genetic determinants of *pbp-1* have been reported to be associated with amoxicillin resistance: S414R and N562Y [51]. Two mutations, S414R and V45I, were present in 67% of amoxicillin-resistant *H. pylori* strains [50,51].

In a study by P. Subsomwong et al. in the Myanmar population, almost all levofloxacin-resistant *H. pylori* isolates had an amino acid substitution at position 91 (Asp-91 to Asn or Tyr). Interestingly, no mutation was identified at position 87, which is associated with fluoroquinolone resistance and is found in levofloxacin-resistant *H. pylori* strains in Myanmar’s neighboring Southeast Asian countries such as Indonesia, Malaysia, and Cambodia. Both mutations are also found in Chinese and Turkish populations [50].

In a large-scale study conducted by Tal Domanovich-Asor et al., the whole genome of *H. pylori* was sequenced (WGS) with the aim of studying the bacterium’s phylogeny and genetic aspects of antibiotic resistance. A total of 1040 genomes of *H. pylori* isolates were analyzed. The study focused on identifying point mutations in genes associated with bacterial antibiotic resistance (*pbp1A, 23S rRNA, gyrA, rdxA, frxA*, and *rpoB*), as well as conducting phylogenetic analysis. As a result, a significant geographic clustering of *H. pylori* genomes was identified in different regions of the world. The resistance analysis showed that the most common point mutations leading to antibiotic resistance were S589G (*pbp1A*, 48.8% of perfectly aligned sequences), A2143G (*23S rRNA*, 27.4% of perfectly aligned sequences), N87 K\I\Y (*gyrA*, 14.7% of perfectly aligned sequences), R131K (*rdxA*, 65.7% of perfectly aligned sequences), and C193S (*frxA*, 62.6% of perfectly aligned sequences). These research results provide a greater understanding of the relationship between antibiotic resistance and changes in the *H. pylori* genome. Further analyses that combine WGS and phenotypic methods will provide a deeper understanding of the relationship between mutations and resistance [60,61].

A group of scientists conducted a 6-year study on the antibiotic susceptibility of *H. pylori* in Israel. The study included 540 *H. pylori* isolates obtained from gastric biopsy specimens, that were collected from 2015 to 2020. Antibiotic resistance to amoxicillin, clarithromycin, metronidazole, levofloxacin, rifampicin, and tetracycline, was evaluated using the E-test method. Generalized linear models were used to estimate differences in gross and adjusted mean MIC values and odds ratios (ORs) for each year compared to the baseline year of 2015, for each antibiotic and for multi-resistance. The results showed the highest resistance rates to clarithromycin and metronidazole: 46.3% and 16.3%, respectively. Patients over 18 years old had higher levels of resistance to rifampicin and multi-resistance (3.3% and 14.8%, respectively) compared to those under 18 years old (0.5% and 8.4%, respectively). Resistance rates to levofloxacin, rifampicin, and multi-resistance were significantly higher among Arab patients compared to Jewish patients. This study highlights the importance of continuous monitoring of *H. pylori* antibiotic resistance for increasing eradication rates of this bacterium. Therapy for *H. pylori* infection should be revisited and updated based on data on antibiotic resistance [62].

A study on the antibiotic resistance of *H. pylori* was conducted in the Tibetan Autonomous Region of China. The study included 397 patients, from whom 153 strains of *H. pylori* were isolated. The overall resistance rates were as follows: clarithromycin (27.4%), levofloxacin (31.3%), metronidazole (86.2%), amoxicillin (15.6%), tetracycline (0%), furazolidone (0.6%), and rifampicin (73.2%). 2.0% of the *H. pylori* isolates were susceptible to all tested antibiotics, with monoresistance, dual resistance, triple resistance, quadruple resistance, and quintuple resistance rates of 18.3%, 44.4%, 18.3%, 12.4%, and 4.6%, respectively. The resistance rates to levofloxacin (40.5%) and amoxicillin (21.5%) in strains obtained from female patients were significantly higher than those in strains obtained from male patients (21.6% and 9.5%, respectively).

The conducted study indicates a very high level of *H. pylori* antibiotic resistance in the Tibetan region of China, which suggests a high risk of developing *Helicobacter*-associated complications in these patients. The high resistance to rifampicin demonstrates the need for further research of its derivative, rifabutin [63].

**Figure 7:**
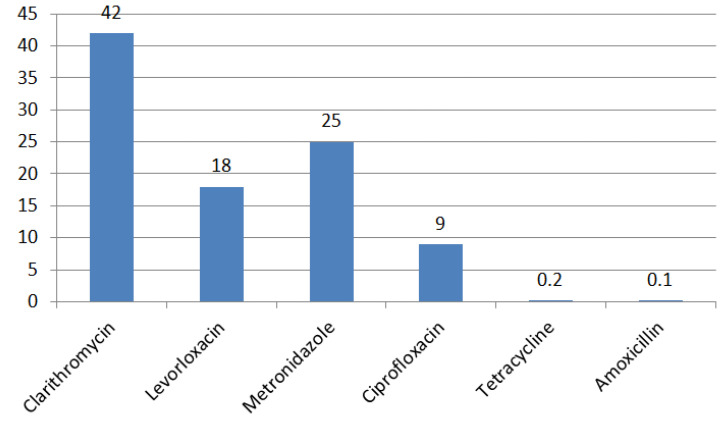
Antibiotic resistance rates (%) of *H. pylori* in Portugal.

A group of scientists from Portugal conducted a meta-analysis of data on *H. pylori* antibiotic resistance in this Country. The analysis included eight cross-sectional studies evaluating the resistance of *H. pylori* to antibiotics. The overall frequency of resistance was as follows: clarithromycin (CLA) 42% (95% CI: 30–54), metronidazole (MTZ) 25% (95% CI: 15–38), ciprofloxacin (CIP) 9% (95% CI: 3–18), levofloxacin (LVX) 18% (95% CI: 2–42), tetracycline (TTC) 0.2% (95% CI: 0–1), and amoxicillin (AMX) 0.1% (95% CI: 0–0.2) (Figure 7). Multiple drug resistance was also evaluated and results for global resistance rates were as follows: CLA plus MTZ 10% (20% in adults (95% CI: 15–26) compared to 6% in children (95% CI: 4–9) and 2% for CLA plus CIP (primary resistance in the pediatric group). High rates of secondary resistance were found for all antibiotics. In relation to antibiotic resistance, the findings indicate that adults exhibited higher levels of resistance to all antibiotics, with the exception of clarithromycin (CLA), which demonstrated high resistance levels in both adults and children (42% 95% CI: 14–71 and 40% 95% CI: 33–47) [64].

A study was conducted to investigate the resistance of *H. pylori* in a cohort of children in Jordan (*n* = 166). The age of the children ranged from 10 to 14 years, with 82.7% of them not having received anti-*Helicobacter* therapy. The authors noted that the detection rate of *H. pylori* infection by rapid urease test, histological, phenotypic, and molecular genetic methods was 93.9%, 89.6%, 61.7%, and 84.3%, respectively. The resistance rates obtained by the phenotypic method were 25.9% for clarithromycin, 50% for metronidazole, and 6.9% for levofloxacin. Interestingly, mutations in the clarithromycin resistance gene were detected in 26.1% of the samples, while mutations in the levofloxacin resistance gene were found in 5.3% of the samples. The authors have concluded that real-time PCR is a valuable alternative method for the identification of *H. pylori* and determination of antibiotic susceptibility [65].

## 5. Conclusions

Eradication therapy is considered as the basis for the elimination of *H. pylori* infection, which leads to a cure for chronic gastritis and a decrease the risk of occurrence and recurrence of erosive and ulcerative lesions of the gastric and duodenal mucosa, prevention of the development and progression of precancerous changes in the gastric mucosa (atrophic gastritis, intestinal metaplasia) and primary prevention of gastric cancer. The main reason for the decrease in the effectiveness of eradication therapy regimens is the formation and increase in *H. pylori* resistance to antibiotics.

*H. pylori* antibiotic resistance is a consequence of the use of ineffective eradication therapy regimens and the widespread use of macrolides and fluoroquinolones for various indications, which leads to the development of corresponding mutations in the *H. pylori* genes. The latest Maastricht VI international consensus recommends individualized prescription of eradication regimens, taking into account antibiotic resistance, as well as empiric therapy, considering also account regional differences in resistance and therapeutic efficacy. These recommendations make it actualize the introduction and increase in the availability of methods for determining the resistance of *H. pylori* to antibiotics, both phenotypic and molecular genetics.

Further investigation of the regional peculiarities of *H. pylori* resistance to antimicrobial agents is crucial, as there are geographical differences in the distribution of bacterial resistance. Accumulation of data on regional features of *H. pylori* antibiotic resistance, assessment of its genetic determinants in combination with the diagnosis of *H. pylori* resistance before the administration anti-*Helicobacter* therapy will make it possible to contain the growth of *H. pylori* antibiotic resistance and increase effectiveness anti-*Helicobacter* therapy.

## Figures and Tables

**Table 1:** Criteria for assessing the sensitivity of *H. pylori* to antimicrobial agents. (in accordance with EUCAST).

Antibiotic	MIC, mg/L	
	Sensitive, ≤	Resistant, >
Amoxicillin	0.25	0.125
Clarithromycin	0.25	0.5
Levofloxacin	1.0	1.0
Tetracycline	1.0	1.0
Metronidazole	8.0	8.0
Rifampicin	1.0	1.0

**Table 2:** Comparison of methods for determining the antibiotic resistance of *H. pylori*.

Method	Sensitivity/Specificity (%)	The Degree of Labor Intensity	Automation Degree	Time Cost
Diffusion method	96/99	High	Low	18–48 h
Serial dilution method	High	High	Low	16–48 h
E-test	96/99	High	Low	16–48 h
PCR method	96/99	Low	High	4–5 h
Direct sequencing	98/98	Medium	High	8–9 h
NGS	98/98	High	Medium	up to 72 h
FISH	High	Medium	High	14–20 h

**Table 3:** Evaluation of genetic resistance of *H. pylori* in world populations.

Region	Number of Samples	Specimen	Methods Used	Antibiotic Type	Gen (Mutation)	Source
Columbia, S. America	*n* = 59	biopsy	PCR, WGS	Amoxicillin Clarithromycin Rifampicin Tetracycline Levofloxacin Metronidazole	WT WT WT *16S rRNA (A926G)* *gyrA (N87I/K, 91G/Y)* *rdxA, frxA*	[48]
Iran	*n* = 82	biopsy	PCR, Sequencing	Clarithromycin	*23S rRNA (A2143G, A2142G)*	[49]
Myanmar	*n* = 150	biopsy	NGS	Metronidazole Amoxicillin Levofloxacin Clarithromycin	*rdxA (V175I, S91P, R16H/C), frxA (L33M)* *pdp1-A (V45I, S414R, V414R, D465K/D, V471H, N564Y)* *gyrA (D91N/G/Y, D210N, K230Q, A524V, A661T)* *gyrB (A584V, N679H, M676V, V614I)* *23S rRNA (T248C)*	[50]
South Korea	*n* = 144	biopsy	PCR, Sequencing	Amoxicillin	*pdp1 (Val16Ile, Val45Ile, Ser414Arg, Asn562Tyr, Thr593Ala, Gly595Ser, Ala599Thr)*	[51]
USA	*n* = 262	biopsy feces	NGS	Amoxicillin Clarithromycin Metronidazole Levofloxacin Tetracycline Rifabutin	*pdp1* *23S rRNA* *rdxA, frxA* *gyrA* *16S rRNA* *rpoB*	[33,34,52]
Tunisia	*n* = 124	biopsy	PCR	Clarithromycin	*23S rRNA (2142G, 2143G)*	[53]
Sudan	*n* = 288	biopsy	PCR	Clarithromycin	*23S rRNA (A2142G, A2143G, T2182C, C2195T)*	[54]
Vietnam	*n* = 185 *n* = 308	biopsy	Sequencing	Clarithromycin Amoxicillin	*23S rRNA (A2142G, A2143G and other point mutations)* *pbp1A (366, 414,473, ins595–596)*	[55] [16]
Russia	*n* = 15	cultures from the biopsy	Sequencing	Clarithromycin Levofloxacin	*23S rRNA (2142G, 2143G)* *gyrA (N87I/K, 91G/Y)*	[56]
Italy	*n* = 95	feces	-	Clarithromycin Levofloxacin	*23S rRNA (A2142G, A2143G)* *gyrA (N87I/K, 91G/Y)*	[57]
China	*n* = 511	cultures from the biopsy	Sequencing	Metronidazole	*rdxA (R16H/C, Y47C, A67V/T, A80T/S, V204I)*	[58]
Bangladesh	*n* = 133	cultures from the biopsy	WGS	Metronidazole Amoxicillin Levofloxacin Clarithromycin	*ribF (D253E), frxA, rdxA, mdaB, omp11*, *pbp1a (N562Y), pbp2 pbp3*, *pbp4* *gyrA (87, 91), gyrB (A343V)* *23S rRNA, infB*	[59]

## Data Availability

Not applicable.

## References

[1] Maev I.V. (2016). Helicobacter Pylori Infection: Monograph.

[2] Mezmale L., Coelho L.G., Bordin D., Leja M. (2020). Epidemiology of *Helicobacter pylori*. Helicobacter.

[3] De Brito B.B., Da Silva F.A.F., Soares A.S., Pereira V.A., Santos M.L.C., Sampaio M.M., Neves P.H.M., De Melo F.F. (2019). Pathogenesis and clinical management of Helicobacter pylori gastric infection. World J. Gastroenterol..

[4] Malfertheiner P., Megraud F., Rokkas T., Gisbert J.P., Liou J.-M., Schulz C., Gasbarrini A., Hunt R.H., Leja M., O’Morain C. (2022). Management of Helicobacter pylori infection: The Maastrix VI/Florence consensus report. Gut.

[5] Tran V., Saad T., Tesfaye M., Walelign S., Wordofa M., Abera D., Desta K., Tsegaye A., Ay A., Taye B. (2022). Analysis of risk factors of Helicobacter pylori (*H. pylori*) and prediction of prevalence: Machine learning approach: Infectious diseases. Gut.

[6] Noto J.M., Rose K.L., Hachey A.J., Delgado A.G., Romero-Gallo J., Wroblewski L.E., Schneider B.G., Shah S.C., Cover T.L., Wilson K.T. (2019). Carcinogenic strains of *Helicobacter pylori* selectively dysregulate the stomach proteome In Vivo, which may be associated with the progression of gastric cancer. Mol. Cell Proteom..

[7] Elbehiry A., Marzouk E., Aldubaib M., Abalkhail F., Anagreyyah S., Anajirih N., Almuzaini A.M., Rawway M., Alfadhel A., Draz A. (2023). *Helicobacter pylori* Infection: Current Status and Future Prospects on Diagnostic, Therapeutic and Control Challenges. Antibiotics.

[8] Kotilea K., Bontems P., Touati E. (2019). Epidemiology, diagnosis and risk factors of *Helicobacter pylori* infection. Adv. Exp. Med. Biol..

[9] Hooi J.K.Y., Lai W.Y., Ng W.K., Suen M.M.Y., Underwood F.E., Tanyingoh D., Malfertheiner P., Graham D.Y., Wong V.W.S., Wu J.C.Y. (2019). Prevalence of *Helicobacter pylori* Infection: Systematic Review and Meta-Analysis. Gastroenterology.

[10] Bordin D., Morozov S., Plavnik R., Bakulina N., Voynovan I., Skibo I., Isakov V., Bakulin I., Andreev D., Maev I. (2022). *Helicobacter pylori* infection prevalence in ambulatory settings in 2017–2019 in Russia: The data of real-world national multicenter trial. Helicobacter.

[11] Puah S.M., Goh K.L., Ng H.K., Chua K.H. (2021). The current state of resistance of Helicobacter pylori to clarithromycin and levofloxacin in Malaysia—Results of a molecular study. PeerJ.

[12] Li Y., Lv T., He C., Wang H., Cram D.S., Zhou L., Zhang J., Jiang W. (2020). Evaluation of multiplex ARMS-PCR for detection of *Helicobacter pylori* mutations conferring resistance to clarithromycin and levofloxacin. Gut Pathog..

[13] Ziver-Sarp T., Yuksel-Mayda P., Saribas S., Demiryas S., Gareayaghi N., Ergin S., Tasci I., Ozbey D., Bal K., Erzin Y. (2021). Point mutations at *gyrA* and *gyrB* genes of levofloxacin resistant *Helicobacter pylori* strains and dual resistance with clarithromycin. Clin. Lab..

[14] Gong M., Han Y., Wang X., Tao H., Meng F., Hou B., Sun B.B., Wang G. (2021). Effect of temperature on metronidazole resistance in *Helicobacter pylori*. Front. Microbiol..

[15] Marais A., Bilardi C., Cantet F., Mendz G.L., Mégraud F. (2003). Characterization of the genes rdxA and frxA involved in metronidazole resistance in *Helicobacter pylori*. Res. Microbiol..

[16] Tran T.T., Nguyen A.T., Quach D.T., Pham D.T.-H., Cao N.M., Nguyen U.T.-H., Dang A.N.-T., Tran M.A., Quach L.H., Tran K.T. (2022). Emergence of amoxicillin resistance and identification of novel mutations of the pbp1A gene in *Helicobacter pylori* in Vietnam. BMC Microbiol..

[17] Tseng Y.-S., Wu D.-C., Chang C.-Y., C-H Kuo C.-H., Yang Y.-C., Jan C.-M., Su Y.-C., Kuo F.-C., Chang L.-L. (2009). Amoxicillin resistance with β-lactamase production in *Helicobacter pylori*. Eur. J. Clin. Investig..

[18] Qureshi N.N., Gallaher B., Schiller N.L. (2014). Evolution of amoxicillin resistance of *Helicobacter pylori* In Vitro: Characterization of resistance mechanisms. Microb Drug Resist..

[19] Kuo C.-G., Ke J.-N., Kuo T., Lin C.-Y., Hsieh S.-Y., Chiu Y.-F., Wu H.-Y., Huang M.-Z., Bui N.-N., Chiu C.-H. (2023). Multiple amino acid substitutions in penicillin-binding protein-1A confer amoxicillin resistance in refractory *Helicobacter pylori* infection. J. Microbiol. Immunol. Infect..

[20] Contreras M., Benejat L., Mujica H., Peña L., García-Amado M.-A., Michelangeli F., Lehours P. (2019). Real-time PCR detection of 16S rRNA-a single mutation of *Helicobacter pylori* isolates associated with a decrease in susceptibility and resistance to tetracycline in the mucous membrane of gastroesophageal individual hosts. J. Med. Microbiol..

[21] Resina E., Gisbert J.P. (2021). Rescue therapy with furazolidone in patients with at least five failures of eradication treatment and multi-resistant *H. pylori* infection. Antibiotics.

[22] Srisuphanunt M., Wilairatana P., Kooltheat N., Duangchan T., Katzenmeier G., Rose J.B. (2023). Molecular Mechanisms of Antibiotic Resistance and Novel Treatment Strategies for *Helicobacter pylori* Infections. Trop. Med. Infect. Dis..

[23] Fauzia K.A., Miftahussurur M., Syam A.F., Waskito L.A., Doohan D., Rezkitha Y.A.A., Matsumoto T., Tuan V.P., Akada J., Yonezawa H. (2020). Biofilm formation and antibiotic resistance phenotype of *Helicobacter pylori* clinical isolates. Toxins.

[24] Miftahussurur M., Fauzia K.A., Nusi I.A., Setiawan P.B., Syam A.F., Waskito L.A., Doohan D., Ratnasari N., Khomsan A., I Ketut Adnyana I.K. (2020). E-test versus agar dilution for antibiotic susceptibility testing of *Helicobacter pylori*: A comparison study. BMC Res. Notes.

[25] Vilaichone R.K., Aumpan N., Ratanachu-Ek T., Uchida T., Tshering L., Mahachai V., Yamaoka Y. (2022). Population-based study of *Helicobacter pylori* infection and antibiotic resistance in Bhutan. Int. J. Infect. Dis..

[26] Raro O.H.F., Collar G.S., Da Silva R.M.C., Vezzaro P., Mott M.P., Da Cunha G.R., Riche C.V.W., Dias C., Caierão J. (2021). Performance of polymyxin B agar-based tests among carbapenem-resistant Enterobacterales. Lett. Appl. Microbiol..

[27] Patel J.B. (2017). Performance Standards for Antimicrobial Susceptibility Testing.

[28] Siavoshi F., Saniee P., Latifi-Navid S., Massarrat S., Sheykholeslami A. (2021). Increased resistance of *H. pylori* isolates to metronidazole and tetracycline—Comparison of three 3-year studies. Arch. Iran. Med..

[29] Rebrikov D.V., Samatov G.A., Trofimov D.Y., Semenov P.A., Savilova A.M., Kofiadi I.A., Abramov I.A. (2019). Real-time PCR-M.

[30] Pichon M., Freche B., Christophe Burucoa C. (2022). New Strategy for the Detection and Treatment of *Helicobacter pylori* Infections in Primary Care Guided by a Non-Invasive PCR in Stool: Protocol of the French HepyPrim Study. J. Clin. Med..

[31] Borodinov A.G., Manoilov V.V., Zarutsky I.V., Petrov A.I., Kurochkin V.E. (2020). Generations of DNA sequencing methods. Sci. Instrum..

[32] Ishibashi F., Suzuki S., Nagai n., Mochida K., Morishita T. (2023). Optimizing *Helicobacter pylori* Treatment: An Updated Review of Empirical and Susceptibility Test-Based Treatments. Gut Liver..

[33] Hulten K.G., Genta R.M., Kalfus I.N., Zhou Y., Zhang H., Graham D.Y. (2021). Comparison of Culture With Antibiogram to Next-Generation Sequencing Using Bacterial Isolates and Formalin-Fixed, Paraffin-Embedded Gastric Biopsies. Gastroenterology.

[34] Moss S.F., Dang L.P., Chua D., Sobrado J., Zhou Y., Graham D.Y. (2022). Comparable Results of *Helicobacter pylori* Antibiotic Resistance Testing of Stools vs Gastric Biopsies Using Next-Generation Sequencing. Gastroenterology.

[35] Celiberto F., Losurdo G., Pricci M., Girardi B., Marotti A., Leo A.D., Ierardi E. (2023). The State of the Art of Molecular Fecal Investigations for *Helicobacter pylori (H. pylori*) Antibiotic Resistances. Int. J. Mol. Sci..

[36] Megraud F., Bruyndonckx R., Coenen S., Wittkop L., Huang T.-D., Hoebeke M., Bénéjat L., Lehours F., Goossens H., Glupczynski Y. (2021). Helicobacter pylori resistance to antibiotics in Europe in 2018 and its relationship to antibiotic consumption in the community. Gut.

[37] Savoldi A., Carrara E., Graham D.Y., Conti M., Tacconelli E. (2018). Prevalence of antibiotic resistance in *Helicobacter pylori*: A systematic review and meta-analysis in World Health Organization regions. Gastroenterology.

[38] Liu Y., Wang S., Yang F., Chi W., Ding L., Liu T., Zhu F., Ji D., Zhou J., Fang Y. (2022). Antimicrobial resistance patterns and genetic elements associated with the antibiotic resistance of *Helicobacter pylori* strains from Shanghai. Gut Pathog..

[39] Cho J.H., Jin S.Y. (2022). Current guidelines for *Helicobacter pylori* treatment in East Asia 2022: Differences am ong China, Japan, and South Korea. World J. Clin. Cases..

[40] Li J., Deng J., Wang Z., Li H., Wan C. (2021). Antibiotic resistance of *Helicobacter pylori* strains isolated from pediatric patients in Southwest China. Front Microbiol..

[41] Shu X., Ye D., Hu C., Peng K., Zhao H., Li H., Jiang M. (2022). Alarming antibiotics resistance of *Helicobacter pylori* from children in Southeast China over 6 years. Sci. Rep..

[42] Andreev D.A., Maev I.V., Kucheryavyy Y.A. (2020). *Helicobacter pylori* resistance in the Russian Federation: A meta-analysis of studies over the past 10 years. Ther. Arch..

[43] Nyssen O.P., Vaira D., Tepes B., Kupcinskas L., Bordin D., Pérez-Aisa A., Gasbarrini A., Castro-Fernández M., Bujanda L., Garre A. (2022). Hp-EuReg Investigators. Room for Improvement in the Treatment of *Helicobacter pylori* Infection: Lessons from the European Registry on *H. pylori* Management (Hp-EuReg). J. Clin. Gastroenterol..

[44] Bujanda L., Nyssen O.P., Vaira D., Saracino L.M., Fiorini G., Lerang F., Georgopoulos S., Tepes B., Heluwaert F., Antonio Gasbarrini A. (2021). The Hp-EuReg Investigators. Antibiotic Resistance Prevalence and Trends in Patients Infected with *Helicobacter pylori* in the Period 2013-2020: Results of the European Registry on *H. pylori* Management (Hp-EuReg). Antibiotics.

[45] Boyanova L., Hadzhiyski P., Gergova R., Markovska R. (2023). Evolution of *Helicobacter pylori* Resistance to Antibiotics: A Topic of Increasing Concern. Antibiotics.

[46] Mégraud F., Alix C., Charron P., Bénéjat L., Ducournau A., Bessède E., Lehours F. (2021). Survey of the antimicrobial resistance of *Helicobacter pylori* in France in 2018 and evolution during the previous 5 years. Helicobacter.

[47] Mosites E., Bruden D., Morris J., Reasonover A., Rudolph K., Hurlburt D., Hennessy T., McMahon B., Bruce M. (2018). Antimicrobial resistance among *Helicobacter pylori* isolates in Alaska, 2000–2016. J. Glob. Antimicrob. Resist..

[48] Mannion A., Dzink-Fox J., Shen Z., Piazuelo M.B., Wilson K.T., Correa P., Peek R.M., Camargo M.C., Fox J.G. (2021). Antimicrobial resistance of *Helicobacter pylori* and gene variants in populations with high and low risk of stomach cancer. J. Clin. Microbiol..

[49] Alavifard H., Mirzaei N., Yadegar A., Baghaei K., Smith S.M., Sadeghi A., Zali M.R. (2021). Investigation of mutations associated with clarithromycin resistance and *Helicobacter pylori* virulence genotypes isolated from the Iranian population: A cross-sectional study. Curr. Microbiol..

[50] Subsomwong P., Doohan D., Fauzia K.A., Akada J., Matsumoto T., Yee T.T., Htet K., Waskito L.A., Tuan V., Uchida T. (2022). Next-Generation Sequencing-Based Study of *Helicobacter pylori* Isolates from Myanmar and Their Susceptibility to Antibiotics. Microorganosms.

[51] Kim B.J., Kim J.G. (2013). Substitutions in penicillin-binding protein 1 in amoxicillin-resistant *Helicoobacter pylori* strains isolated from Korean patients. Gut Liver..

[52] De Palma G.Z., Mendiondo N., Wonaga A., Viola L., Ibarra D., Campitelli E., Salim N., Corti R., Goldman C., Catalano M. (2017). Occurrence of mutations in the antimicrobial target genesrelated to levofloxacin, clarithromycin, and amoxicillin resistance in *Helicobacter pylori* isolates from Buenos Aires city. Microb. Drug Resist..

[53] Chtourou L., Moalla M., Mnif B., Smaoui H., Gdoura H., Boudabous M., Mnif L., Amouri A., Hammami A., Tahri N. (2022). Prevalence of *Helicobacter pylori* resistance to clarithromycin in Tunisia. J. Med. Microbiol..

[54] Albasha A.M., Elnosh M.M., Osman E.H., Zeinalabdin D.M., Fadl A.A.M., Ali M.A., Altayb H.N. (2021). *Helicobacter pylori 23S rRNA* gene A2142G, A2143G, T2182C, and C2195T mutations associated with clarithromycin resistance detected in Sudanese patients. BMC Microbiol..

[55] Tran V.H., Ha T.M.T., Le P.T.Q., Phan T.N., Tran T.N.H. (2019). Characterisation of point mutations in domain V of the *23S rRNA* gene of clinical *Helicobacter pylori* strains and clarithromycin-resistant phenotype in central Vietnam. J. Glob. Antimicrob. Resist..

[56] Tsapkova L.A., Polyakova V.V., Bodunova N.A., Baratova I.V., Voynovan I.N., Dekhnich N.N., Ivanchik N.V., Sabelnikova E.A., Bordin D.S. (2022). Possibilities of a molecular genetic method for detecting resistance to clarithromycin and levofloxacin in *Helicobacter pylori*. Eff. Pharmacother..

[57] Iannone A., Giorgio F., Russo F., Riezzo G., Girardi B., Pricci M., Palmer S.C., Barone M., Principi M., Strippoli G.F. (2018). New fecal test for non-invasive *Helicobacter pylori* detection: A diagnostic accuracy study. Clin. Trial.

[58] Gong Y., Zhai R., Sun L., He L., Wang H., Guo Y., Zhang J. (2023). RdxA Diversity and Mutations Associated with Metronidazole Resistance of *Helicobacter pylori*. Microbiol. Spectr..

[59] Fauzia K.A., Aftab H., Tshibangu-Kabamba E., Alfaray R.I., Saruuljavkhlan B., Cimuanga-Mukanya A., Matsumoto T., Subsomwong P., Akada J., Miftahussurur M. (2023). Bangladesh—Mutations Related to Antibiotics Resistance in *Helicobacter pylori* Clinical Isolates from Bangladesh. Antibiotics.

[60] Domanovich-Asor T., Craddock H.A., Motro Y., Khalfin B., Peretz A., Moran-Gilad J. (2021). Unraveling antimicrobial resistance in *Helicobacter pylori*: Global resistome meets global phylogeny. Helicobacter.

[61] Domanovich-Asor T., Motro Y., Khalfin B., Craddock H.A., Peretz A., Moran-Gilad J. (2021). Genomic Analysis of Antimicrobial Resistance Genotype-to-Phenotype Agreement in *Helicobacter pylori*. Microorganisms.

[62] Azrad M., Vazana D., On A., Paritski M., Rohana H., Roshrosh H., Agay-Shay K., Peretz A. (2022). Antibiotic resistance patterns of *Helicobacter pylori* in North Israel—A six-year study. Helicobacter.

[63] Tang X., Wang Z., Shen Y., Song X., Benghezal M., Marshall B.J., Tang H., Hong Li H. (2022). Antibiotic resistance patterns of *Helicobacter pylori* strains isolated from the Tibet Autonomous Region, China. Microbiology.

[64] Lopo I., Libânio D., Pita I., Dinis-Ribeiro M., Pimentel-Nunes P. (2018). *Helicobacter pylori* antibiotic resistance in Portugal: Systematic review and meta-analysis. Helicobacter.

[65] Lee Y.-C., Dore M.P., Graham D.Y. (2022). Diagnosis and treatment of *Helicobacter pylori* infection. Annu. Rev. Med..

